# Increasing utilization of intrauterine device insertion at hysteroscopic endometrial evaluation for patients with endometrial hyperplasia

**DOI:** 10.1007/s00404-024-07411-7

**Published:** 2024-03-22

**Authors:** Katharine M. Ciesielski, Pavan K. Mann, Rachel S. Mandelbaum, Maximilian Klar, Lynda D. Roman, Jason D. Wright, Koji Matsuo

**Affiliations:** 1 Department of Minimally Invasive Gynecologic Surgery, Department of Obstetrics and Gynecology, University of Michigan, Ann Arbor, MI USA; 2 Division of Gynecologic Oncology, Department of Obstetrics and Gynecology, University of Southern California, 2020 Zonal Avenue, IRD 520, Los Angeles, CA 90033 USA; 3 Division of Reproductive Endocrinology & Infertility, Department of Obstetrics and Gynecology, University of Southern California, Los Angeles, CA USA; 4 Department of Obstetrics and Gynecology, Freiburg Faculty of Medicine, University of Freiburg, Freiburg, Germany; 5 Norris Comprehensive Cancer Center, University of Southern California, Los Angeles, CA USA; 6 Division of Gynecologic Oncology, Department of Obstetrics and Gynecology, Columbia University School of Medicine, New York, NY USA

**Keywords:** Endometrial hyperplasia, Hysteroscopic endometrial resection, Intrauterine device insertion, Ambulatory, Same day surgery

## Abstract

**Purpose:**

To examine the utilization and characteristics related to the use of hysteroscopy at the time of endometrial evaluation for endometrial hyperplasia in the outpatient surgery setting.

**Methods:**

This cross-sectional study queried the Healthcare Cost and Utilization Project’s Nationwide Ambulatory Surgery Sample. The study population was 3218 patients with endometrial hyperplasia who underwent endometrial evaluation from January 2016 to December 2019. Performance and clinical characteristics of hysteroscopic endometrial evaluation were assessed with multivariable binary logistic regression models.

**Results:**

A total of 2654 (82.5%) patients had hysteroscopic endometrial tissue evaluation. Patients with postmenopausal bleeding, heavy menstrual bleeding, and polycystic ovary syndrome were more likely to undergo hysteroscopic endometrial evaluation in multivariable analysis (all, adjusted-*P* < 0.001). Uterine injury occurred in 4.9 per 1000 hysteroscopic endometrial evaluations; none had uterine injury in the non-hysteroscopy cohort. Among the 2654 patients who had hysteroscopic endometrial evaluation, 106 (4.0%) patients had intrauterine device insertion at surgery, and the utilization increased from 2.9 to 5.8% during the study period (*P-trend* < 0.001). Younger age, more recent year surgery, and obesity were independently associated with increased utilization of intrauterine device insertion at hysteroscopic endometrial evaluation (all, adjusted-*P* < 0.05). Among 2023 reproductive-age patients with endometrial hyperplasia, 1666 (82.4%) patients underwent hysteroscopic endometrial evaluation. On multivariable analysis, patients with heavy menstrual bleeding were more likely to have hysteroscopic endometrial evaluation (adjusted-*P* < 0.05). Intrauterine device insertion increased from 3.7% in 2016 to 8.0% in 2019 (*P-trend* = 0.007).

**Conclusion:**

This nationwide analysis suggests that the insertion of intrauterine devices at the time of hysteroscopic endometrial tissue evaluation for endometrial hyperplasia is increasing among reproductive-age population.

**Supplementary Information:**

The online version contains supplementary material available at 10.1007/s00404-024-07411-7.

## What does this study add to the clinical work

**Table:** 

1. Hysteroscopy appears to be frequently incorporated into the endometrial evaluation of patients with endometrial hyperplasia in the recent years in the USA. 2. Intrauterine device insertion at the time of hysteroscopic endometrial tissue evaluation for endometrial hyperplasia is increasing in reproductive-age population.	

## Introduction

Endometrial hyperplasia is a premalignant precursor of endometrial cancer, characterized by disorganized proliferative endometrial glands, and is usually the result of unopposed estrogen exposure [1]. Endometrial carcinoma is the most common gynecologic malignancy in the USA [2], and the incidence of endometrial hyperplasia is estimated to be four to seven times higher than that of endometrial cancer [3].

Accurate diagnosis of endometrial hyperplasia is paramount to inform patient management. First, there is a risk of progression from endometrial hyperplasia to endometrial cancer (1–3% without atypia and 8–29% with atypia) [4, 5]. Second, occult endometrial cancer can co-exist with endometrial hyperplasia. In atypical endometrial hyperplasia, the incidence of occult endometrial cancer found on post-hysterectomy specimens is nearly 40% [6]. More importantly, the reproducibility of the diagnosis of endometrial hyperplasia with atypia across pathologists is poor and adenocarcinoma is often underestimated [7]. This is particularly important for reproductive-age patients who desire fertility-sparing treatment.

The common diagnostic approach for endometrial hyperplasia is endometrial tissue evaluation. While not required, hysteroscopic evaluation at the time of endometrial curettage was recommended by the American College of Obstetricians and Gynecologists (ACOG) in 2015 to identify any discrete endometrial lesions and to improve the sensitivity of histologic evaluation of endometrial hyperplasia [8]. A 2016 meta-analysis of 27 studies examining 1,106 patients concluded that hysteroscopic endometrial resection reduces the under-diagnosis of endometrial cancer [9]. A 2023 single center study further suggested utility for tumor differentiation evaluation [10]. However, conflicting data exist regarding the concern of disseminating precancerous or cancerous cells into the peritoneal cavity with the use of hysteroscopic distension fluids. [11, 12]

To date, national-level practice patterns describing the use of hysteroscopic endometrial evaluation for endometrial hyperplasia are understudied in the USA. The objective of the current study was to describe the utilization and characteristics related to hysteroscopy use at the time of endometrial evaluation for endometrial hyperplasia in the ambulatory surgery setting in the USA.

## Material and methods

### Data source

This cross-sectional study queried the Healthcare Cost and Utilization Project’s Nationwide Ambulatory Surgery Sample (NASS) through the Agency for Healthcare Research and Quality [13]. NASS is the largest all-payer database for ambulatory surgery in the USA. The NASS program approximates a stratified-sample of 67% of ambulatory surgery performed in each hospital-owned facility every year. In 2019, nearly 9 million encounters, estimating 11.8 million encounters for national-level statistics, were collected across 2958 facilities [13]. The University of Southern California Institutional Review Board deemed this study exempt due to the use of publicly available, deidentified data.

### Study population and exposure assignment

The study population was patients with a diagnosis of endometrial hyperplasia, both with and without atypia, who underwent endometrial evaluation in the ambulatory surgery setting from January 2016 to December 2019. The diagnosis of endometrial hyperplasia was based on the World Health Organization’s International Classification of Disease 10th revision (ICD-10) code of N85.0 (Supplemental Table S1) [14]. Patients with a diagnosis of gynecologic malignancy (uterine cancer, cervical cancer, and ovarian cancer) were excluded from the analysis.

The surgical procedures for endometrial evaluation were based on the American Medical Association’s Current Procedural Terminology (CPT) codes. Specifically, endometrial evaluation with uterine curettage alone was based on the CPT codes of 58,120, 58,100, and 58,110, and hysteroscopic endometrial evaluation was based on the CPT codes of 58,558 (Supplemental Table S1). The infrequent cases that had the CPT codes for both hysteroscopy and uterine curettage or endometrial evaluation were also considered hysteroscopic endometrial evaluation in this study. The data-sampling mechanism did not capture the cases of office-based endometrial biopsy.

### Outcome measures

The primary outcome measure was the performance and clinical characteristics of hysteroscopic endometrial evaluation. The secondary outcome measures included surgical morbidity and intrauterine device insertion at the time of endometrial evaluation.

### Study variables

Among the patients eligible for analysis, patient demographics, gynecologic information, surgical information, hospital parameters, and surgical complications were abstracted from the NASS program (a total of 23 preselected covariates). The ICD-10 and CPT codes for the study variables are shown in Supplemental Table S1. These codes were unchanged throughout the study period.

Patient demographics included age at surgery (≤ 49 and ≥ 50 years) dichotomized per the upper cutoff for reproductive-age definition per the World Health Organization, year of encounter (2016, 2017, 2018, and 2019), primary expected payer (Medicare, Medicaid, private including HMO, self-pay, no charge, and other defined by the program), census-level median household income (every quartile), patient location (large central metropolitan, large fringe metropolitan, medium metropolitan, small metropolitan, micropolitan, and not metropolitan or micropolitan counties), obesity (yes or no), and Charlson comorbidity index (0, 1, 2, and ≥ 3) [15]. These were selected to assess whether hysteroscopy choice differs per baseline patient demographic.

Gynecologic information included the diagnosis of abnormal uterine bleeding, postmenopausal bleeding, heavy menstrual bleeding, scant rare menstruation, polycystic ovary syndrome, infertility, uterine myoma, uterine adenomyosis, uterine anomaly, and endometrial hyperplasia type (non-atypia or atypia). These were chosen to examine whether the choice of hysteroscopic evaluation is based on functional, structural, or histological factors.

Surgical information detailed concurrent procedures at the time of endometrial evaluation, such as intrauterine device insertion, diagnostic laparoscopy, and total hysterectomy. Hospital parameters included hospital bed capacity (small, mid, and large), hospital location and teaching setting (rural, urban non-teaching, and urban teaching), and hospital region (Northeast, Midwest, South, and West). These were evaluated based on the assumption that availability and access to surgical equipment may differ across hospital factors. Surgical morbidity evaluated in this study included uterine injury and fluid or electrolyte abnormalities.

### Analytic approach

The first step of analysis was to describe the utilization rate of hysteroscopic endometrial evaluation across the study covariates and to identify the independent characteristics associated with hysteroscopic endometrial evaluation for patients with endometrial hyperplasia. A multivariable binary logistic regression model was fitted for analysis, and parsimonious, conditional backward selection was used for the covariate selection. In the initial modeling, all the study covariates with *P* < 0.05 level in univariable analysis were selected. These variables were chosen because they could be known risk factors, potential confounders, or variables of interest in understanding the utilization of hysteroscopic endometrial evaluation in patients with endometrial hyperplasia. In the following steps, least significant factor was sequentially excluded from the analysis with the stopping rule of *P* < 0.05 in the final modeling [16]. This approach was chosen due to relatively modest sample size and to avoid overfitting. The effect size for hysteroscopic endometrial evaluation compared to endometrial curettage alone was expressed with adjusted-odds ratio (aOR) with a corresponding 95% confidence interval (CI).

The second step of analysis was to evaluate the performance of intrauterine device insertion at the time of endometrial evaluation for endometrial hyperplasia. The annual temporal trend of intrauterine device insertion was assessed with the Cochran-Armitage trend test from 2016 to 2019. A multivariable logistic regression model with backward selection method was used for the final covariate selection. This stepwise analysis was performed in each exposure stratum.

Sensitivity analyses included evaluation of hysteroscopic endometrial evaluation in reproductive-age patients with endometrial hyperplasia. This subgroup was chosen as these patients may be fertility-sparing candidates where conservative approach with intrauterine device insertion may be more favored in recent years. [17]

The weighted values for national estimates provided by the NASS program were utilized for statistical analysis. Statistical interpretation was based on a two-tailed hypothesis, and a* P* < 0.05 was considered statistically significant. Cases with missing information were grouped as one category in each variable. Sample size estimation was not performed due to the nature of population-level, nationwide assessment. IBM SPSS Statistics (version 28.0, Armonk, NY, USA) was used for all analyses. The STROBE reporting guidelines were followed to summarize the performance of the study. [18]

## Results

### Cohort-level analysis

A total of 3218 patients with a diagnosis of endometrial hyperplasia underwent endometrial evaluation in the ambulatory surgery setting from 2016 to 2019 for national estimates. The median age was 44 (interquartile range 35–56) years, and 62.9% of the study population were of reproductive-age of ≤ 49 years. The majority of patients were privately insured (66.0%) and had the surgery in large (51.0%), urban teaching (62.5%) facilities (Table 1). Approximately 2% of patients underwent hysterectomy following endometrial evaluation during the encounter (2.1%).

**Table 1:** Use of hysteroscopy (all-age cohort)

Characteristic	No.^a^	Hystero (%)^b^	*P*-value
No	3,218 (100)	82.5	
Age (y)			0.815
≤ 49	2,023 (62.9)	82.4	
≥ 50	1,195 (37.1)	82.7	
Year			0.279*
2016	880 (27.3)	81.3	
2017	722 (22.4)	83.1	
2018	808 (25.1)	82.1	
2019	808 (25.1)	83.7	
Primary expected payer			0.063
Medicare	430 (13.4)	83.3	
Medicaid	495 (15.4)	81.0	
Private including HMO	2,123 (66.0)	83.1	
Self-pay	74 (2.3)	68.9	
No charge	**	**	
Other^c^	90 (2.8)	82.2	
Unknown	**	**	
Household income			0.059
QT1 (lowest)	807 (25.1)	80.5	
QT2	860 (26.7)	85.6	
QT3	797 (24.8)	82.3	
QT4 (highest)	710 (22.1)	81.4	
Unknown	45 (1.4)	77.8	
Patient location			< 0.001
Large central metropolitan	850 (26.4)	75.6	
Large fringe metropolitan	807 (25.1)	85.4	
Medium metropolitan	662 (20.6)	84.7	
Small metropolitan	309 (9.6)	87.4	
Micropolitan	312 (9.7)	83.3	
Not metropolitan or micropolitan	277 (8.6)	83.0	
Unknown	**	**	
Obesity			0.637
No	2,440 (75.8)	82.3	
Yes	778 (24.2)	83.0	
Charlson comorbidity index			0.694
0	2,376 (73.8)	82.5	
1	570 (17.7)	83.0	
2	194 (6.0)	79.4	
≥ 3	79 (2.5)	83.5	
Abnormal uterine bleeding			0.067
No	2,727 (84.7)	82.0	
Yes	492 (15.3)	85.4	
Postmenopausal bleeding			< 0.001
No	2,820 (87.6)	81.5	
Yes	399 (12.4)	89.2	
Heavy menstrual bleeding			< 0.001
No	2,550 (79.2)	81.1	
Yes	668 (20.8)	87.7	
Scant rare menstruation			0.534
No	3,178 (98.7)	82.4	
Yes	41 (1.3)	87.8	
Polycystic ovary syndrome			0.014
No	3,034 (94.3)	82.1	
Yes	184 (5.7)	89.1	
Infertility			0.064
No	3,143 (97.7)	82.3	
Yes	75 (2.3)	90.7	
Uterine myoma			0.191
No	2,739 (85.1)	82.8	
Yes	479 (14.9)	80.4	
Adenomyosis			0.280
No	3,144 (97.7)	82.3	
Yes	75 (2.3)	88.0	
Uterine anomaly			0.047
No	3,190 (99.1)	82.4	
Yes	28 (0.9)	96.4	
Histology type			0.232
Non-atypia	1,232 (38.3)	83.9	
Atypia	438 (13.6)	81.5	
NOS	1,548 (48.1)	81.6	
Intrauterine device insertion			0.108
No	3,081 (95.7)	82.7	
Yes	137 (4.3)	77.4	
Diagnostic laparoscopy			0.493
No	3,203 (99.5)	82.7	
Yes	15 (0.5)	93.3	
Hysterectomy			0.007
No	3,151 (97.9)	82.7	
Yes	67 (2.1)	70.1	
Hospital bed capacity			0.025
Small	419 (13.0)	83.3	
Mid	1,159 (36.0)	84.6	
Large	1,640 (51.0)	80.7	
Hospital location/teaching			0.148
Rural	416 (12.9)	84.4	
Urban non-teaching	790 (24.5)	84.1	
Urban teaching	2,012 (62.5)	81.5	
Hospital region			< 0.001
Northeast	573 (17.8)	82.0	
Midwest	891 (27.7)	85.5	
South	1,184 (36.8)	83.0	
West	571 (17.7)	77.1	

Total number may not be 3,218 due to weighted value

*Hystero* hysteroscopic endometrial sampling, *QT* quartile, *NOS* not otherwise specified

^a^Percentage per column

^b^Percentage per row

^c^Primary payer types were grouped per the HCUP and other group included payer types that were not itemized in the table

*Cochran-Armitage trend test

**Small number suppressed per HCUP guidelines

Of the study population, 2654 (82.5%) patients had hysteroscopic endometrial evaluation while 564 (17.5%) patients had endometrial curettage alone without hysteroscopy. On univariable analysis (Table 1), hysteroscopic endometrial evaluation was associated with a number of patient factors including residential location, gynecologic factors including heavy menstrual bleeding, postmenopausal bleeding, polycystic ovary syndrome, and uterine anomaly, hospital factors including hospital bed capacity and facility region, and surgical factors including hysterectomy arrangement (all, *P* < 0.05). Histologic subtypes of endometrial hyperplasia were not associated with hysteroscopy use (*P* = 0.232).

In multivariable analysis (Table 2), patients in the large central metropolitan area were less likely to have hysteroscopic endometrial evaluation compared to those in large fringe metropolitan area (aOR 0.59, 95% CI 0.46–0.77). Patients with heavy menstrual bleeding (aOR 1.79, 95% CI 1.38–2.31), postmenopausal bleeding (aOR 2.14, 95% CI 1.52–3.00), and polycystic ovary syndrome (aOR 1.69, 95% CI 1.05–2.71) were more likely to undergo hysteroscopic endometrial evaluation. Patients undergoing endometrial evaluation at centers in the Midwest were more likely to undergo hysteroscopic procedures (aOR 1.53, 95% CI 1.15–2.03). Patients undergoing hysteroscopic endometrial evaluation were less likely to have a hysterectomy during the same encounter compared to those undergoing endometrial evaluation without hysteroscopy (aOR 0.45, 95% CI 0.26–0.78).

**Table 2:** Multivariable analysis for hysteroscopic endometrial sampling (all-age cohort)

Characteristic	aOR (95%CI)	*P*-value
Patient location		< 0.001*
Large central metropolitan	0.59 (0.46–0.77)	< 0.001
Large fringe metropolitan	1.00 (ref)	
Medium metropolitan	0.94 (0.70–1.25)	0.659
Small metropolitan	1.13 (0.76–1.67)	0.551
Micropolitan	0.87 (0.61–1.25)	0.452
Not metropolitan or micropolitan	0.79 (0.55–1.16)	0.229
Unknown	n/a	0.999
Postmenopausal bleeding		
No	1.00 (ref)	
Yes	2.14 (1.52–3.00)	< 0.001
Heavy menstrual bleeding		
No	1.00 (ref)	
Yes	1.79 (1.38–2.31)	< 0.001
Polycystic ovary syndrome		
No	1.00 (ref)	
Yes	1.69 (1.05–2.71)	0.031
Hysterectomy		
No	1.00 (ref)	
Yes	0.45 (0.26–0.78)	0.004
Hospital region		0.034*
Northeast	1.24 (0.92–1.67)	0.162
Midwest	1.53 (1.15–2.03)	0.003
South	1.29 (1.00–1.66)	0.051
West	1.00 (ref)	

A binary logistic regression model for multivariable analysis (conditional backward method with stopping rule of *P* < 0.05)

*aOR* adjusted-odds ratio, *CI* confidence interval, *ref* reference

*Overall *P*-value

The incidence rate of any measured adverse events was 5.0 per 1,000 patients at the cohort level evaluation. Of which, the majority were uterine injury (81.3%). The uterine injury rate was 4.9 per 1000 in the hysteroscopic endometrial evaluation group. None of the patients who underwent endometrial curettage without hysteroscopy had uterine injury.

### Intrauterine device insertion

**Fig. 1:**
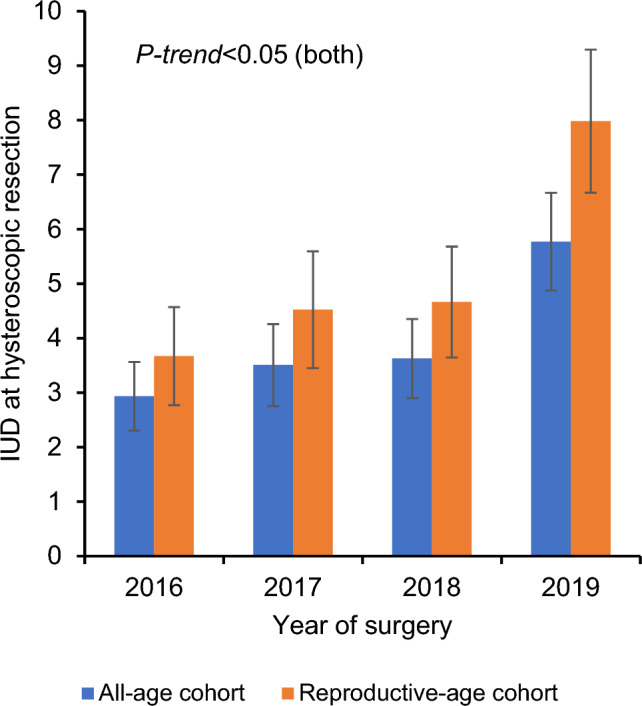
Trends of IUD insertion at hysteroscopic endometrial evaluation. Cochrane-Armitage trend test for *P*-value. Error bars indicate standard error. *IUD* intra-uterine device insertion

Among the 2,654 patients who had hysteroscopic endometrial evaluation, 106 (4.0%) patients had intrauterine device insertion at the time of surgery. The performance of intrauterine device insertion increased from 2.9% in 2016 to 5.8% in 2019 during the study period (*P-trend* < 0.001; Fig. 1).

On univariable analysis (Table 3), intrauterine device insertion was associated with younger age, year of surgery, patient location, obesity, abnormal uterine bleeding, heavy menstrual bleeding, postmenopausal bleeding, histology, and hospital region (all, *P* < 0.05). In a multivariable analysis (Table 4), reproductive-age patients (aOR 3.70, 95% CI 2.20–6.23), those who had surgery more recently (aOR for 2019 compared to 2016, 1.90, 95% CI 1.10–3.31), and obese patients (aOR 1.64, 95% CI 1.08–2.47) were more likely to undergo intrauterine device insertion. The utilization of intrauterine device insertion was similar for those who underwent endometrial curettage without hysteroscopy (*P-trend* = 0.467).

**Table 3:** IUD insertion at hysteroscopic endometrial sampling

Characteristic	No.^a^	IUD (%)^b^	*P*-value
No	2,654	4.0	
Age (y)			< 0.001
≤ 49	1,666 (62.8)	5.3	
≥ 50	988 (37.2)	1.8	
Year			0.010*
2016	715 (27.0)	2.9	
2017	600 (22.6)	3.5	
2018	663 (25.0)	3.6	
2019	676 (25.5)	5.8	
Primary expected payer			0.059
Medicare	358 (13.5)	**	
Medicaid	401 (15.1)	4.7	
Private including HMO	1,764 (66.5)	4.2	
Self-pay	51 (1.9)	**	
No charge	**	0	
Other	74 (2.8)	**	
Unknown	**	0	
Household income			0.542
QT1 (lowest)	650 (24.5)	3.2	
QT2	736 (27.7)	4.3	
QT3	656 (24.7)	4.1	
QT4 (highest)	578 (21.8)	4.5	
Unknown	35 (1.3)	0	
Patient location			0.034
Large central metropolitan	643 (24.2)	6.2	
Large fringe metropolitan	689 (25.9)	3.2	
Medium metropolitan	561 (21.1)	3.2	
Small metropolitan	270 (10.2)	**	
Micropolitan	260 (9.8)	5.0	
Not metropolitan or micropolitan	230 (8.7)	**	
Unknown	**	0	
Obesity			< 0.001
No	2,008 (75.7)	3.2	
Yes	646 (24.3)	6.5	
Charlson comorbidity index			0.170
0	1,961 (73.9)	3.7	
1	473 (17.8)	4.9	
2	154 (5.8)	**	
≥ 3	66 (2.5)	**	
Abnormal uterine bleeding			< 0.001
No	2,235 (84.2)	3.4	
Yes	420 (15.8)	6.9	
Postmenopausal bleeding			0.013
No	2,299 (86.6)	4.3	
Yes	356 (13.4)	**	
Heavy menstrual bleeding			0.045
No	2,068 (77.9)	4.4	
Yes	586 (22.1)	2.6	
Scant rare menstruation			0.400
No	2,619 (98.7)	4.0	
Yes	36 (1.3)	0	
Polycystic ovary syndrome			0.534
No	2,490 (93.8)	3.9	
Yes	164 (6.2)	**	
Infertility			0.752
No	2,586 (97.4)	4.0	
Yes	68 (2.6)	**	
Uterine myoma			0.916
No	2,269 (85.5)	4.0	
Yes	385 (14.5)	3.9	
Adenomyosis			0.999
No	2,589 (97.5)	4.0	
Yes	66 (2.5)	**	
Uterine anomaly			0.091
No	2,627 (99.0)	3.9	
Yes	27 (1.0)	**	
Histology type			< 0.001
Non-atypia	1,034 (39.0)	5.3	
Atypia	357 (13.5)	7.8	
NOS	1,263 (47.6)	1.7	
Diagnostic laparoscopy			0.999
No	2,640 (99.5)	4.0	
Yes	14 (0.5)	0	
Hysterectomy			0.260
No	2,607 (98.2)	4.1	
Yes	47 (1.8)	0	
Hospital bed capacity			0.205
Small	349 (13.2)	5.7	
Mid	981 (37.0)	3.7	
Large	1,324 (49.9)	3.8	
Hospital location/teaching			0.578
Rural	351 (13.2)	3.1	
Urban non-teaching	664 (25.0)	3.8	
Urban teaching	1,639 (61.8)	4.3	
Hospital region			0.012
Northeast	470 (17.7)	3.0	
Midwest	762 (28.7)	4.1	
South	983 (37.0)	3.2	
West	440 (16.6)	6.6	

*QT* quartile, *NOS* not otherwise specified

^a^Percentage per column

^b^Percentage per row

*Cochrane-Armitage trend test

**Small number suppressed per HCUP guidelines. Total number may not be 2654 due to weighted value

**Table 4:** Multivariable analysis for IUD insertion at hysteroscopic endometrial sampling

Characteristic	aOR (95% CI)	*P*-value
Age		
≤ 49	3.70 (2.20–6.23)	< 0.001
≥ 50	1.00 (ref)	
Year		0.044*
2016	1.00 (ref)	
2017	1.00 (0.54–1.87)	0.990
2018	1.15 (0.63–2.11)	0.649
2019	1.90 (1.10–3.31)	0.022
Obesity		
No	1.00 (ref)	
Yes	1.64 (1.08–2.47)	0.020
Heavy menstrual bleeding		
No	1.00 (ref)	
Yes	0.46 (0.26–0.80)	0.007
Histology type		< 0.001*
Non-atypia	1.00 (ref)	
Atypia	1.41 (0.87–2.29)	0.162
NOS	0.30 (0.18–0.50)	< 0.001

A binary logistic regression model for multivariable analysis (conditional backward method with stopping rule of *P* < 0.05)

*aOR* adjusted-odds ratio, *CI* confidence interval, *ref* reference

*Overall *P*-value

### Reproductive-age cohort

The performance of hysteroscopic endometrial evaluation was examined in 2023 patients who were of reproductive-age at the time of surgery. The median age was 37 years (interquartile range 31–43), and 38.6% were aged < 35 years. Nearly quarter were obese (25.0%). Abnormal uterine bleeding and heavy menstrual bleeding were seen in 20.5 and 29.8% of patients, respectively (Supplemental Table S2). Polycystic ovarian syndrome and uterine myoma were reported in 8.9 and 14.0% of the study population, respectively.

A total of 1666 (82.4%) patients had the endometrial evaluation with hysteroscopy guidance. On univariable analysis (Supplemental Table S2), hysteroscopic endometrial evaluation was associated with primary expected payer, patient location, heavy menstrual bleeding, polycystic ovary syndrome, and hospital region (all, *P* < 0.05). In multivariable analysis (Supplemental Table S3), patients with heavy menstrual bleeding (aOR 1.79, 1.35–2.37) were more likely to have hysteroscopic endometrial evaluation whereas self-pay patients, those in large central metropolitan areas, and Western U.S. residents were less likely.

Following hysteroscopic endometrial evaluation, intrauterine device insertion was performed in 5.2% of reproductive-age patients. Over time, the performance of intrauterine device insertion increased from 3.7% in 2016 to 8.0% in 2019 (*P-trend* = 0.007; Fig. 1).

## Discussion

### Principal findings

This analysis of hysteroscopic endometrial evaluation for patients with endometrial hyperplasia revealed a number of important findings. First, hysteroscopy is frequently performed at the time endometrial evaluation. Second, insertion of intrauterine devices at the time of hysteroscopic endometrial evaluation for endometrial hyperplasia is increasing, particularly among reproductive-age patients. Given scarcity in data that analyzed this practice on a national-level, the current study adds new information to the literature.

### Insights for results

#### Clinical implications

Various factors were associated with U.S. surgeons’ decision to incorporate hysteroscopy at the time of endometrial evaluation for endometrial hyperplasia. Patients with endometrial hyperplasia in the setting of postmenopausal bleeding were more likely to undergo hysteroscopic evaluation compared to endometrial curettage alone which may be due to a higher index of suspicion for underlying malignancy. PCOS is a known risk factor for endometrial pathology, increasing a woman’s lifetime risk of endometrial malignancy up to three-fold [19]. This may be why patients with PCOS were also more likely to undergo hysteroscopic endometrial evaluation.

Structural causes of heavy menstrual bleeding, such as polyps or myoma, can be both identified and removed via hysteroscopy, which may explain the higher utilization of hysteroscopy for patients with heavy menstrual bleeding compared to endometrial curettage without hysteroscopy. Interestingly, there was no difference in hysteroscopy use between those with or without atypia in this study, which suggests that other factors play into the determination of which patient will undergo hysteroscopic endometrial evaluation.

Recent studies have shown that endometrial cancer in reproductive-age women is gradually increasing [20], and this may also be true for its precursor, endometrial hyperplasia. As intrauterine device insertion at the time of hysteroscopic endometrial evaluation increased over time among reproductive-age patients observed in this national-level assessment, this may reflect this growing patient population’s desire for fertility-sparing treatment options. In the reproductive-age population there is a nationwide increase in the utilization of intrauterine device therapy for endometrial hyperplasia with atypia in the USA and this trend may possibly occur in non-atypia. [17]

Obesity was also associated with an increased use of intrauterine device insertion at the time of hysteroscopic endometrial evaluation in this study, which may be due to the improved efficacy of a progestin-releasing intrauterine device over systemic progestins [21], or to avoid more invasive surgery in future, such as a hysterectomy [17], in a higher risk patient population.

While there was an increased risk of uterine injury in the hysteroscopic endometrial evaluation cohort compared to those who did not undergo hysteroscopy, the rate of complications was lower than that reported in previously published data [22, 23]. For example, a 2002 French study reported the uterine perforation rate of 1.6% (95%CI 1.1–2.1). It may be possible that evolving surgical technique and device over decades contributed the decreasing procedure-related morbidity [22]. In addition, the risk of uterine injury may be underestimated in the non-hysteroscopy cohort given the lack of direct visualization.

#### Research implications

This study identified characteristics associated with the incorporation of hysteroscopy at the time of endometrial evaluation; however, additional data, such as ultrasound findings or previous treatment with medications known to increase the risk of endometrial cancer, could further elucidate surgeons’ decisions to perform endometrial evaluation with or without hysteroscopy.

Future studies are also necessary to determine whether the use of hysteroscopy has an impact on patient outcomes, such as progression to endometrial cancer or overall survival. Hysteroscopy use has been associated with increased risk of malignant peritoneal cytologic washings [11, 12], and while this factor was removed from the staging system of endometrial cancer in 2009, multiple societies and organizations recommend evaluation of the peritoneal cytology at hysterectomy and recent evidence shows that malignant peritoneal cytology may be associated with decreased survival. [24, 25]

### Study limitations

There are several limitations in the current study. First, unmeasured bias is inherent in any retrospective study. Possible confounders that were not captured in the study but may influence the analysis included performance of office-based endometrial biopsy prior to outpatient surgical evaluation, preoperative diagnosis for surgery (*e.g.*, known diagnosis of endometrial hyperplasia), surgeon type (gynecologic oncologist or gynecologist), and shared decision-making process with patient. It is paramount to be aware that these unmeasured confounders could potentially affect research outcomes.

Second, selection bias may exist in this study as the cases that had office-based biopsy only including sono-hysterogram or office hysteroscopy, which ACOG recommends for the evaluation and treatment of endometrial polyps when available [26], without performance of a procedure in an ambulatory surgical setting evaluation, were not captured in the NASS program. It is likely that a large number of patients with endometrial hyperplasia only had office-based endometrial evaluation with endometrial biopsy.

Third, lack of information on final pathologic information for occult endometrial cancer including incidence per evaluation modality as well as peritoneal cytology status is crucial in this study as these are key outcome measures for this type of study [11, 12]. It may be possible that patients in the hysteroscopic resection group may have a higher detection rate of occult endometrial cancer compared to the patients in the non-hysteroscopy group [9]. The impact of this information on subsequent treatment can be possibly significant. For instance, diagnosis of endometrial cancer vs endometrial hyperplasia may influence the shared decision-making in reproductive-age patients when proceeding fertility-sparing approach. Among patients with preoperative endometrial hyperplasia who undergo definitive surgical treatment with hysterectomy, possible opportunity to evaluate sentinel lymph node may be missed when occult endometrial cancer is not detected in preoperative endometrial sampling. [27]

Fourth, specific type of intrauterine device was not available in the coding schema. Fifth, accuracy of data in the NASS program was not assessable without actual medical record review. In addition, CPT codes were not distinguishable for hysteroscopy-guided direct resection of endometrial lesion *versus* biopsy. Sixth, due to the coding schema, chorology of measured procedures was not assessable in the study. Seventh, menopause data, past pregnancy history, and desire of future fertility were not available due to lack of coding schema but these may possibly impact treatment approach. Reproducibility of study results by external investigators was not assessed in the study. Last, although several results showed statistical significance, its clinical significance and utility may be limited.

## Conclusions

Given that hysteroscopic guidance may be used in the majority of patients undergoing endometrial evaluation in the ambulatory setting in the USA in recent years, more studies to examine the risks and benefits of this surgical procedure would be useful to address aforementioned research questions. This is particularly applicable for the concurrent intrauterine device insertion at the time of hysteroscopic endometrial evaluation that the practice has increased over time as fertility-sparing surgery for reproductive-aged patients. [28, 29]

### Supplementary Information

Below is the link to the electronic supplementary material.

Supplementary file1 (PDF 136 KB)

## Data Availability

The data on which this study is based are publicly available upon request at Healthcare Cost and Utilization Project, Agency for Healthcare Research and Quality. https://www.hcup-us.ahrq.gov/nassoverview.jsp.

## Declarations

### Transparency

The manuscript’s corresponding author (K.M.) affirms that the manuscript is an honest, accurate, and transparent account of the study being reported; that no important aspects of the study have been omitted; and that any discrepancies from the study as planned (and, if relevant, registered) have been explained. The Nationwide Ambulatory Surgery Sample is a part of the Healthcare Cost and Utilization Project by the Agency for Healthcare Research and Quality and is the source of the de-identified data used; and the program has not verified and is not responsible for the statistical validity of the data analysis or the conclusions derived by the study team.
