# Supplementary material for: Increasing utilization of intrauterine device insertion at hysteroscopic endometrial evaluation for patients with endometrial hyperplasia
Source: Arch Gynecol Obstet. 2024 Mar 22;309(6):2709–18. doi: 10.1007/s00404-024-07411-7 (PMC11147815; doi:10.1007/s00404-024-07411-7)
Supplement: Supplementary file 1 — Supplementary file1 (PDF 136 KB) [file 404_2024_7411_MOESM1_ESM.pdf]

Supplemental Table S1. Coding information.

Characteristic	ICD-10-CM	CPT
Endometrial hyperplasia	N85.00, N85.01,* N85.02†	
Hysteroscopic resection		58558, 58555 with 58120
Uterine curettage, endometrial sampling		58120, 58100, 58110
Obesity	E660, E661, E662, E668, E669, O9921	
AUB	N93	
PMB	N95.0	
Heavy menstrual bleeding	N92	
Scant rare menstruation	N91	
PCOS	E28.2	
Infertility	N97	
Uterine myoma	D25	
Adenomyosis	N80.0	
Uterine anomaly	Q51	
IUD	Z30430	58300
Diagnostic laparoscopy		49320
Hysterectomy		58150, 58152, 58200, 58210; 58548, 58570, 58571, 58572, 58573, 58575; 58550, 58552, 58553, 58554, 58553; 58260, 58262, 58263, 58267, 58270, 58275, 58290, 58291, 58292, 58293, 58294, 58285
Uterine injury	S37.6	
Electrolytes abnormality	E87	

The program provided clinical factors not listed in this table as discrete study variables. *endometrial hyperplasia without atypia. †endometrial hyperplasia with atypia. Abbreviations: ICD-10-CM, International Classification of Disease, 10th revision, Clinical Modification; CPT, Current Procedural Terminology; AUB, abnormal uterine bleeding; PMB, postmenopausal bleeding; PCOS, polycystic ovary syndrome; and IUD, intrauterine device insertion.

Supplemental Table S2. Use of hysteroscopy (reproductive-age cohort).

Characteristic	No.†	Hystero (%)§	*P*-value
No.	2023	82.4	
**Age (y)**			0.330
<35	781 (38.6)	83.4	
≥35	1243 (61.4)	81.7	
**Year**			0.931*
2016	534 (26.4)	81.6	
2017	443 (21.9)	84.9	
2018	532 (26.3)	80.6	
2019	515 (25.4)	82.7	
**Primary expected payer**			0.002
Medicare	58 (2.9)	69.0	
Medicaid	414 (20.5)	81.2	
Private including HMO	1431 (70.8)	83.9	
Self-pay	60 (3.0)	66.7	
No charge	**	**	
Other	56 (2.8)	80.4	
Unknown	**	**	
**Household income**			0.824
QT1 (lowest)	574 (28.4)	81.4	
QT2	555 (27.4)	84.0	
QT3	468 (23.1)	82.3	
QT4 (highest)	401 (19.8)	81.8	
Unknown	26 (1.3)	80.8	
**Patient location**			<0.001
Large central metropolitan	553 (27.3)	76.3	
Large fringe metropolitan	481 (23.8)	86.7	
Medium metropolitan	413 (20.4)	82.8	
Small metropolitan	183 (9.0)	84.7	
Micropolitan	214 (10.6)	86.0	
Not metropolitan or micropolitan	178 (8.8)	81.5	
Unknown	**	**	
**Obesity**			0.219
No	1518 (75.0)	81.8	
Yes	505 (25.0)	84.2	
**Charlson comorbidity index**			0.556
0	1572 (77.7)	82.7	
1	345 (17.1)	82.0	
2	81 (4.0)	76.5	
≥3	25 (1.2)	84.0	
**Abnormal uterine bleeding**			0.139
No	1608 (79.5)	81.7	
Yes	415 (20.5)	84.8	
**Postmenopausal bleeding**			0.149
No	2009 (99.3)	82.2	
Yes	14 (0.7)	100	
**Heavy menstrual bleeding**			<0.001
No	1420 (70.2)	80.0	
Yes	603 (29.8)	87.9	
**Oligomenorrhea**			0.533
No	1983 (98.0)	82.2	
Yes	41 (2.0)	87.8	

**Polycystic ovary syndrome**			0.015
No	1843 (91.1)	81.7	
Yes	181 (8.9)	89.0	
**Infertility**			0.063
No	1948 (96.3)	82.0	
Yes	75 (3.7)	90.7	
**Uterine myoma**			0.097
No	1739 (86.0)	82.9	
Yes	284 (14.0)	78.9	
**Adenomyosis**			0.235
No	1961 (96.9)	82.2	
Yes	62 (3.1)	88.7	
**Uterine anomaly**			0.156
No	2001 (98.9)	82.2	
Yes	22 (1.1)	95.5	
**Histology type**			0.474
Non-atypia	761 (37.6)	82.7	
Atypia	250 (12.4)	79.6	
NOS	1012 (50.0)	82.8	
**Intrauterine device insertion**			0.974
No	1917 (94.7)	82.4	
Yes	107 (5.3)	82.2	
**Diagnostic laparoscopy**			0.999
No	2017 (99.7)	82.4	
Yes	**	**	
**Hysterectomy**			0.999
No	2002 (98.9)	84.0	
Yes	22 (1.1)	81.8	
**Hospital bed capacity**			0.392
Small	253 (12.5)	84.2	
Mid	795 (39.3)	83.1	
Large	977 (48.2)	81.2	
**Hospital location / teaching**			0.956
Rural	285 (14.1)	82.8	
Urban non-teaching	505 (25.0)	82.6	
Urban teaching	1233 (60.9)	82.2	
**Hospital region**			<0.001
Northeast	329 (16.3)	83.0	
Midwest	566 (28.0)	85.9	
South	769 (38.0)	83.0	
West	360 (17.8)	75.0	

† Percentage per column. § Percentage per row. * Cochrane-Armitage trend test. ** Small number suppressed per HCUP guidelines. Total number may not be 2,023 due to weighted value. Abbreviations: QT, quartile; and NOS, not otherwise specified.

Supplemental Table S3. Multivariable analysis for hysteroscopic endometrial sampling (reproductive-age cohort).

Characteristic	aOR (95%CI)	*P*-value
**Primary expected payer**		0.005*
Medicare	0.43 (0.23-0.81)	0.009
Medicaid	1.00 (ref)	
Private including HMO	1.15 (0.86-1.54)	0.344
Self-pay	0.48 (0.26-0.89)	0.019
No charge	n/a	0.999
Other	0.91 (0.44-1.87)	0.788
Unknown	n/a	0.999
**Patient location**		<0.001*
Large central metropolitan	0.56 (0.40-0.79)	<0.001
Large fringe metropolitan	1.00 (ref)	
Medium metropolitan	0.72 (0.50-1.05)	0.088
Small metropolitan	0.83 (0.51-1.36)	0.471
Micropolitan	0.98 (0.60-1.58)	0.919
Not metropolitan or micropolitan	0.59 (0.37-0.95)	0.029
Unknown	n/a	0.999
**Heavy menstrual bleeding**		
No	1.00 (ref)	
Yes	1.79 (1.35-2.37)	<0.001
**Hospital region**		*0.008
Northeast	1.50 (1.02-2.20)	0.040
Midwest	1.82 (1.27-2.59)	<0.001
South	1.52 (1.11-2.09)	0.009
West	1.00 (ref)	

A binary logistic regression model for multivariable analysis (conditional backward method with stopping rule of *P*<0.05). *Overall *P*-value. Abbreviations: aOR, adjusted-odds ratio; CI, confidence interval; and ref, reference.
